# Mental health needs in war-affected refugee children: barriers, gaps, and strategies for effective care

**DOI:** 10.1186/s13034-024-00840-x

**Published:** 2024-11-13

**Authors:** Mohsen Khosravi

**Affiliations:** 1 Department of Psychiatry, School of Medicine, Zahedan University of Medical Sciences, Zahedan, 9813913777 Iran; 2 Health Promotion Research Center, Zahedan University of Medical Sciences, Zahedan, Iran; 3 Community Nursing Research Center, Zahedan University of Medical Sciences, Zahedan, Iran

**Keywords:** Children, Mental health, Needs, Refugees, War

## Abstract

War-affected refugee children often experience significant disruptions to their mental health due to exposure to traumatic events, displacement, and the challenges of resettlement. This comprehensive overview examines the substantial mental health needs of these children and identifies the barriers and gaps that hinder effective mental health care delivery. The study highlights the critical aspects of mental health requirements, including the impact of trauma and displacement, and explores the systemic obstacles that prevent adequate access to care. By addressing these barriers and gaps, the paper aims to inform strategies for improving mental health services for war-affected refugee children, ultimately contributing to better mental health outcomes for this vulnerable population.

War-affected refugee children constitute one of the most vulnerable groups worldwide, facing significant disruptions to their mental health due to exposure to traumatic events, displacement, and the complexities of resettlement [1]. The mental health needs of this group of people are multifaceted and severe. These children often endure a range of traumatic experiences, including witnessing violence, losing family members, and being forcibly displaced from their homes [2]. Such experiences can lead to a variety of psychological issues, including post-traumatic stress disorder, depression, anxiety, adjustment disorders, psychosomatic symptoms, behavioral problems (e.g., aggression, withdrawal, and difficulties in social interactions), suicidality, and developmental delays [3–6]. These young refugees, who have often fled from conflict zones, face not only the trauma of their past experiences but also new hurdles in their host countries. One of the most pressing issues they encounter is bullying, which can take many forms, including verbal harassment, physical intimidation, sexual violence and abuse, and social exclusion [7–9]. In schools, where refugee children are expected to integrate and thrive academically and socially, they often find themselves targets of bullying due to their different backgrounds, languages, or cultures. This can exacerbate feelings of isolation and fear, making it difficult for them to adjust to their new environment [8]. The impact of such bullying is profound; it can lead to anxiety, depression, and a decline in academic performance [10]. In response to these hostile environments, refugee children frequently form tight-knit groups within schools as a means of protection and solidarity. These groups offer a sense of belonging and security amidst an otherwise challenging setting. However, forming these protective circles can sometimes lead to further alienation from other students who may perceive them as insular or unapproachable. This dynamic can inadvertently perpetuate cycles of misunderstanding and prejudice between refugee children and their peers [8, 10, 11]. Moreover, the process of displacement itself introduces additional stressors. Refugee children frequently face uncertainty about their future, separation from loved ones, and the loss of community and cultural identity. These factors, combined with severe financial difficulties, uncertain asylum status, and overt personal racism, contribute to a widespread sense of instability and insecurity, exacerbating existing mental health issues and creating new ones [2–6, 12, 13]. Another layer of complexity for war-affected refugee children is the generational conflicts that arise within their families [14]. Many young refugees take on significant responsibilities as interpreters for their families even at very young ages. This role reversal places them in positions where they must navigate complex bureaucratic systems or medical appointments while translating for parents who may not yet speak the host country’s language fluently. Such responsibilities can place immense pressure on these children, burdening them with adult-like duties that exceed their developmental stage. These experiences can lead to tension within families as traditional roles are disrupted; parents may feel disempowered while children grapple with stress and confusion about their place within both family dynamics and broader society [15]. Therefore, it is essential for support systems—such as community organizations and mental health services—to recognize these unique challenges faced by refugee families [14, 15].

Altogether, the cumulative effect of these traumas (both those resulting from past experiences and those arising from new challenges in host countries) can severely impact war-affected refugee children’s emotional well-being, cognitive development, and social functioning, which requires special attention from healthcare and mental health providers [3–9]. Despite the critical need for mental health services, war-affected refugee children encounter numerous barriers that prevent them from receiving adequate care [16]. One significant barrier is the lack of awareness and understanding of mental health issues within refugee communities. Cultural stigmas surrounding mental health can discourage families from seeking help, as they may fear judgment or ostracism. Language barriers also pose a significant challenge. Many refugee children and their families may not speak the language of their host country fluently, making it difficult to communicate their needs and understand available resources. This language gap can lead to misunderstandings and misdiagnoses, further complicating the provision of appropriate care. Additionally, there is often a shortage of mental health professionals trained to address the unique needs of refugee children. Many host countries lack sufficient resources and specialized services to support this population adequately. Even when services are available, they may not be accessible due to logistical issues such as transportation difficulties or long waiting lists [16–19].

The gaps in mental health services for war-affected refugee children are significant and multifaceted. One major gap is the lack of integrated care models that address both the psychological and practical needs of these children [20]. Effective mental health care for refugee children should ideally include not only therapeutic interventions but also support for education, social integration, and family reunification [21]. Another gap is the insufficient focus on culturally sensitive approaches. Mental health interventions must be tailored to the cultural backgrounds and experiences of refugee children to be effective. However, many existing programs do not adequately consider these factors, leading to interventions that may not resonate with or fully support the children they aim to help [22]. Furthermore, there is a need for more robust data collection and research on the mental health outcomes of war-affected refugee children. Without comprehensive data, it is challenging to develop evidence-based policies and programs that effectively address their needs. Improved research efforts could help identify best practices and inform the development of more targeted and effective interventions [23–25]. Table 1 presents a detailed examination of the mental health barriers and gaps experienced by war-affected refugee children, alongside a comprehensive set of strategies aimed at addressing these challenges [16–26]. This table serves as a crucial resource for mental health professionals, policymakers, educators, and humanitarian workers who are dedicated to improving the well-being of this vulnerable population.

In conclusion, the mental health needs of war-affected refugee children are profound and complex, requiring urgent attention and action. By addressing the barriers and gaps through targeted strategies, we can make significant strides in improving the mental health outcomes for war-affected refugee children. It is essential to adopt a holistic approach that considers the multifaceted nature of their experiences and needs. Collaboration among international organizations, local governments, non-profits, and community leaders is vital to ensure that these children receive the comprehensive support they deserve.

## Data Availability

No datasets were generated or analysed during the current study.
