# Correction: Mental health needs in war-affected refugee children: barriers, gaps, and strategies for effective care

**DOI:** 10.1186/s13034-024-00857-2

**Published:** 2025-02-25

**Authors:** Mohsen Khosravi

**Affiliations:** 1 Department of Psychiatry, School of Medicine, Zahedan University of Medical Sciences, Zahedan, 9813913777 Iran; 2 Health Promotion Research Center, Zahedan University of Medical Sciences, Zahedan, Iran; 3 Community Nursing Research Center, Zahedan University of Medical Sciences, Zahedan, Iran

**Correction to: Child and Adolescent Psychiatry and Mental Health (2024) 18:146**

10.1186/s13034-024-00840-x

In the original publication of this article [1], Table 1 was missing from this article; the Table 1 should have appeared as shown below.

**Table 1:** Mental health barriers and gaps, and strategies for addressing them among war-affected refugee children [16–26]

Barriers and gaps to mental health care
• Stigma: Mental health issues are often stigmatized within refugee communities, leading to reluctance in seeking help • Language and cultural differences: Language barriers and cultural differences can impede communication between refugees and mental health professionals • Lack of resources: Refugee camps and host countries may lack the necessary resources and infrastructure to provide adequate mental health services • Trauma-informed care: A lack of training in trauma-informed care among healthcare providers can result in inadequate or inappropriate treatment • Legal and policy barriers: Refugee children may face legal and policy barriers that restrict their access to mental health services. This includes issues related to asylum status, documentation, and healthcare policies in host countries. Physical access to mental health services can also be challenging due to geographical, financial, or security constraints
**Strategies for addressing mental health barriers and gaps**
• Early intervention: - Implement screening programs to identify children at risk of mental health issues early on - Provide immediate psychological first aid and support to children upon arrival in refugee camps or host countries • Culturally sensitive care: - Train mental health professionals in cultural competence to understand and respect the cultural backgrounds of refugee children - Employ interpreters and cultural mediators to bridge language gaps and facilitate effective communication • Community involvement: - Engage community leaders and members in mental health awareness campaigns to reduce stigma and encourage help-seeking behavior - Establish peer support groups where children can share their experiences and support each other • Integrated services: - Develop integrated care models that combine mental health services with primary healthcare, education, and social services - Collaborate with non-governmental organizations and international agencies to pool resources and expertise • Capacity building: - Invest in training programs for healthcare providers on trauma-informed care and child-specific mental health interventions, such as those focused on verbal processing of past experiences, a variety of creative art techniques, or a combination of these approaches - Strengthen the mental health infrastructure in host countries to ensure sustainable support for refugee children - Collaborate with international organizations to provide technical support and resources • Specialized therapeutic services: - Develop mobile mental health clinics to reach remote or underserved areas - Utilize telehealth services to connect children with mental health professionals when face-to-face interaction is not possible. • Policy advocacy: - Advocate for policies that prioritize mental health funding in humanitarian aid budgets - Work with governments and international bodies to create frameworks that ensure sustained mental health support for refugee children • Ensure sustainable funding: - Securing long-term funding for providing consistent and reliable mental health care • School-based programs: - Integrate psychosocial support into the school curriculum through activities that promote emotional resilience and coping skills - Provide training for teachers to recognize signs of mental distress and refer students to appropriate services - provide resources such as language support programs and anti-bullying initiatives in schools
